# Construction, purification, and characterization of a chimeric TH1 antagonist

**DOI:** 10.1186/1472-6750-6-25

**Published:** 2006-05-22

**Authors:** Iraldo Bello-Rivero, Yeny Torrez-Ruiz, Elizabeth Blanco-Garcés, Giselle Pentón-Rol, Osmani Fernández-Batista, Luís Javier-González, Haydee Gerónimo-Perez, Pedro López-Saura

**Affiliations:** 1 Clinical Trial Department, Center for Biological Research, Calle 134 entre 23 y 25, Cubanacan, P.O. Box 6332, Havana, Cuba; 2 Physicochemical Division, Center for Genetic Engineering and Biotechnology, Calle 190 entre 31 y 33. Postal Code 10600, Havana, Cuba; 3 Department of Biologicals, Control Quality Division, Center for Genetic Engineering and Biotechnology, Calle 190 entre 31 y 33. Postal Code 10600, Havana, Cuba

## Abstract

**Background:**

TH1 immune response antagonism is a desirable approach to mitigate some autoimmune and inflammatory reactions during the course of several diseases where IL-2 and IFN-γ are two central players. Therefore, the neutralization of both cytokines could provide beneficial effects in patients suffering from autoimmune or inflammatory illnesses.

**Results:**

A chimeric antagonist that can antagonize the action of TH1 immunity mediators, IFN-γ and IL-2, was designed, engineered, expressed in *E. coli*, purified and evaluated for its in vitro biological activities. The TH1 antagonist molecule consists of the extracellular region for the human IFNγ receptor chain 1 fused by a four-aminoacid linker peptide to human 60 N-terminal aminoacid residues of IL-2. The corresponding gene fragments were isolated by RT-PCR and cloned in the pTPV-1 vector. *E. coli *(W3110 strain) was transformed with this vector. The chimeric protein was expressed at high level as inclusion bodies. The protein was partially purified by pelleting and washing. It was then solubilized with strong denaturant and finally refolded by gel filtration. In vitro biological activity of chimera was demonstrated by inhibition of IFN-γ-dependent HLA-DR expression in Colo 205 cells, inhibition of IFN-γ antiproliferative effect on HEp-2 cells, and by a bidirectional effect in assays for IL-2 T-cell dependent proliferation: agonism in the absence versus inhibition in the presence of IL-2.

**Conclusion:**

TH1 antagonist is a chimeric protein that inhibits the in vitro biological activities of human IFN-γ, and is a partial agonist/antagonist of human IL-2. With these attributes, the chimera has the potential to offer a new opportunity for the treatment of autoimmune and inflammatory diseases.

## Background

Interferon gamma (IFN-γ), produced by activated T and NK cells [1], macrophages and dendritic cells [2], has important immunomodulatory and inflammatory actions [3,4]. The activities of IFN-γ are initiated following association of the cytokine with a membrane-bound receptor (IFNGR) present on many cell types [5]. The receptor comprises two subunits (IFNGR1 and IFNGR2). IFNGR1 has an extracellular portion of 228 residues [6], that also occurs in soluble form and can function as an endogenous IFN-γ inhibitor [7]. Several pathological effects have been ascribed to IFN-γ in animal models and in humans. IFN-γ neutralization inhibits the lethal effect of endotoxin in an animal model of septic shock [8], as well as the rejection of tumor, skin, and heart allografts [9,10]. IFN-γ-mediates pancreatic beta-cell death and the subsequent development of immune-mediated diabetes [11,12] and accelerates the development of lupus-like disease and nephritis in NZW × NZB- after treatment of F1 mice, whereas antibodies to IFN-γ can block or delay the progression of the disease [13,14]. Additionally, administration of IFN-γ can promote the development of reactive gliosis in the central nervous system (CNS) of adult mice [15] and aggravate the course of multiple sclerosis in humans [16]. These observations suggest that IFN-γ antagonist may have therapeutic application in autoimmune diseases, chronic inflammation, and allograft rejection. Recently, an anti-IFNγ antibody (Fontolizumab) has been demonstrated to be of a clinical benefit in patients suffering from Crohn's disease [17].

Interleukin 2 (IL-2) is a lymphokine synthesized and secreted primarily by T-helper lymphocytes that have been activated by stimulation with certain mitogens or by interaction of the T-cell receptor complex with an antigen/MHC complex on the surfaces of antigen-presenting cells [18]. The biological activities of IL-2 are mediated through its binding to a multisubunit cellular receptor. Although three distinct transmembrane glycoprotein subunits contribute to the formation of the "definitive" high affinity IL-2 receptor, various combinations of receptor subunits are known to occur [19,20]. Resting cells do not express high-affinity IL-2R, but activation with antigen rapidly [21] induces expression.

The main non-redundant activity of IL-2 consists in the regulation of T-cell tolerance [22] and along with IFN-γ and TNF-β, it is a defining product of the TH1 subset. Production of IL-2 may contribute to the pathogenesis of some diseases: overproduction of IL-2 has been seen in patients with multiple sclerosis [23-25], systemic lupus erythematosus relapses [26], myasthenia gravis [27] and psoriasis [28]. However, recent studies indicate that failure of CD4 (+) CD25 (+) regulatory T cells to develop is the underlying cause of autoimmunity in the absence of IL-2 [22]. These observations indicating that both IFN-γ and IL-2 can promote pathogenesis of inflammation, prompted us to develop a chimeric protein TH1 antagonist that can simultaneously modulate the biological activities of both cytokines.

## Results and discussion

### Construction and expression of TH1 antagonist

The poly-A mRNA for each nucleic acid to be cloned was amplified from Jurkat and Raji cells expressing IL-2 and high levels of IFNGR1, respectively. cDNAs for hu IL-2 N-terminal fragment (coding for the first 60 aminoacid residues) and IFNGR1 subunit extracellular region (coding for 228 amino acids) were isolated by RT-PCR amplification of isolated poly-A mRNA. The cloning strategy included amplification of each cDNA fragment using primers with overlapping nucleotides to permit fusion of the two bands in a later second round-PCR. The fragments were joined in a second PCR using the 5' primer from IL-2 fragment amplification and the 3' primer for IFNGR1 extracellular fragment first-round amplification.

**Figure 1:**
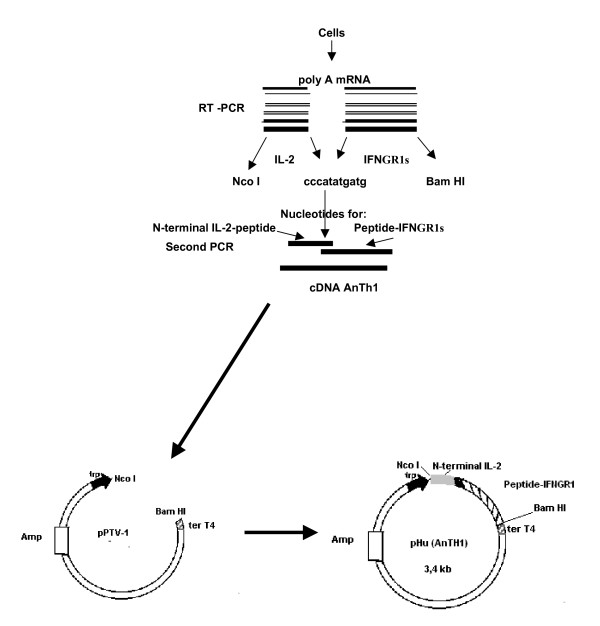
**Construction of the expression plasmid pHu (AnTH1)**. The figure illustrates the process started from Poly-A mRNA purification from cell lines, followed by the RT-PCR of respective cDNA fragments. The amplified cDNA TH1 antagonist (AnTh1) contains in its extremes the sequences for *NcoI *and *BamHI *restriction enzymes suited for cloning in the pPTV-1 vector (left at the bottom of diagram). In the center is indicated the nucleotide sequence (ccatatgatg) coding for the linking peptide. The final genetic construction is shown at the bottom right.

These primers contain the sequences suited for *Nco I *and *Bam HI *restriction enzyme cuts, compatible with respective restriction sites in the pTPV-1 expression vector, in which the final fused cDNA was inserted. The resulting vector contains a *BamH*I site just before the translational stop codon and the cloned fragment is denoted pHu (AnTH1). Figure 1 summarizes the strategy followed to obtain the expression vector pHu (AnTH1).

### Expression and purification of AnTH1

**Figure 2:**
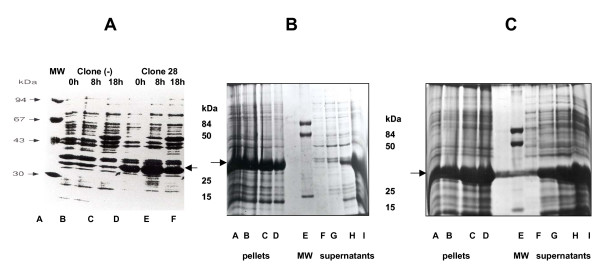
***E coli *expression and urea extraction of recombinant chimeric protein**. Panel A: Coomasie staining of 12.5% SDS-PAGE in reduced conditions of samples from *E coli *expression. *Lanes A, B, C*: *E coli *strain negative control (empty vector), 0, 8 and 18 hours of induction; *Lanes D, E, F*: *E. coli *strains containing the expression plasmid pHu (AnTH1) under induction conditions: 0, 8, and 18 hours of induction. Panel B: *Urea extraction*. Coomasie staining of 12.5% SDS-PAGE under reducing conditions. *Lanes A, B, C, and D*, pellets and *lanes F, G, H, I*, supernatants from extraction with 0, 2, 4 and 8 mol/L urea. Panel C: *Lanes A, B, C, and D*, pellets and *lanes F, G, H, I*, supernatants *from *extraction with 0, 5, 6 and 7 mol/L urea. *Lanes E *in panels B and C indicate the position of MW. The samples contain approximately the same amount of protein. The arrows indicate the position of AnTh1 recombinant protein

W3110 P3 *E. coli *cells were transformed with pHu (AnTH1) plasmid incubated in 500 mL growth medium yielded about 1 g of *E. coli *wet biomass, in which TH1 antagonist protein constituted about 30 % of the total protein (Figure 2A). A small quantity of TH1 antagonist was expressed at the start of the induction period, likely related to the fact that pTrip is a strong promoter difficult to be completely silenced. In the same figure, the lanes corresponding to the negative control show a band much fainter with the same migration rate as that of the recombinant molecule. This band was not observed after purification steps and therefore probably represents a co-migrating *E. coli *protein.

Since expression in *E. coli *culture resulted in accumulation of the recombinant protein in insoluble "inclusion bodies", purification of expressed chimera was performed by washing the pellets using a low urea concentration, followed by a high urea concentration for the extraction (solubilization) processes. Comparative studies showed that 8 mol/L urea solubilized approximately the 78% of recombinant protein (see Table 1); lower concentrations of urea were less efficient (Figure. 2B and 2C) and were used for the washing steps. This approach achieves a good performance; since in the washed pellet the insoluble recombinant protein is retained while soluble *E. coli *proteins are washed out, providing a high grade of purification at the end of the process.

**Figure 3:**
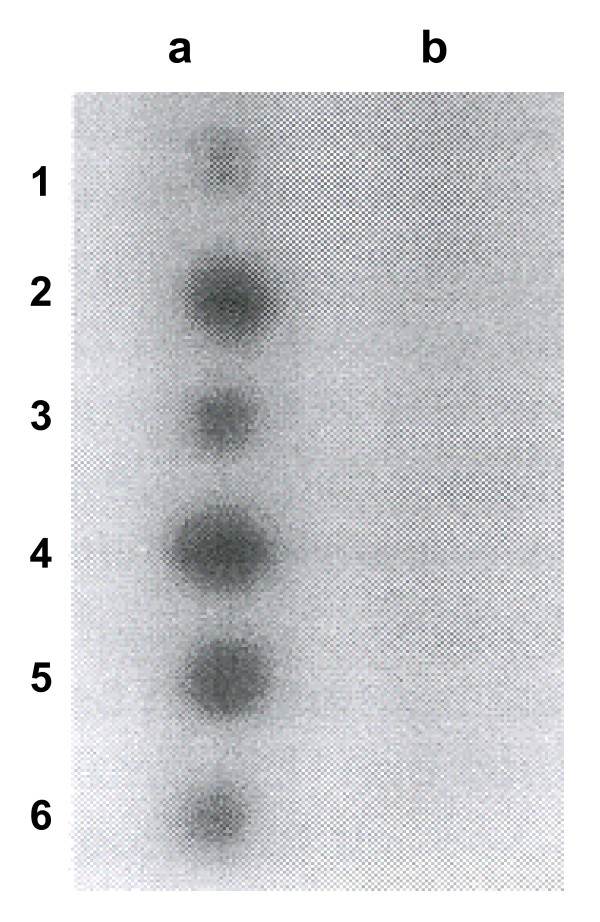
**Binding assay of rhu ^125^I^-^IFN-γ labeled to recombinant chimeric protein using dot blot analysis**. 1 μl aliquots of the eluted fractions from the gel filtration chromatography (*rows 1, 2, 3, 4, 5, 6*) were applied to nitrocellulose membrane strips. The strips were incubated with IFN-γ labeled with ^125^I (35 μCi/μg) in the absence (*lane a*) and in the presence (*lane b*) of 100 fold excess of human IFN-γ.

**Figure 4:**
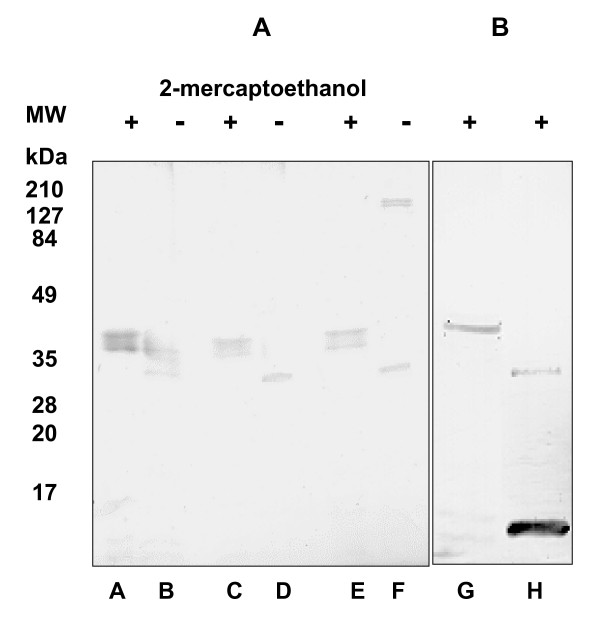
**Electrophoretic mobility of recombinant chimeric protein in reduced and non-reduced conditions**. Panel A: The antagonist was identified in a Western blot using a rabbit anti-IL-2 polyclonal antibody. The analyzed TH1 antagonist preparations were those obtained from the 8 M urea extraction, (*lanes a and b*), gel filtration fraction in 4 M urea (*lanes c and d*) and after dialysis against PBS (*lanes e and f*). Panel B: Western blot to identify the TH1 antagonist after dialysis against PBS (*lane g*) and IL-2 (*lane h*) in reduced conditions using the anti-IL-2 antibody.

During the refolding process, realized by moving to a mild denaturating agent and finally to a physiological buffer, the reduced denatured protein recovers its active conformation, as demonstrated by protein ligand blots, where the active soluble chimera specifically bound radiolabed hu recombinant IFN-γ (Figure 3), as well as by biological activity tests (see the next section). The main recombinant product is a 33 kDa or 37 kDa protein in non-reduced or reduced conditions, respectively, as recognized by anti-IL-2 polyclonal antibody (Figure 4A). Shift in mobility of TH1 antagonist between non-reducing and reduced eletrophoretic conditions is in correspondence with what has been reported for the soluble IFNGR [29] and for several other receptors [30,31] and is consistent with a more compact structure of folded protein. Polymeric and monomeric forms were detected, probably as a consequence of various alternative possibilities in the formation of disulfide bonds among the nine sulphydryl groups of the protein [32]. The anti-IL-2 polyclonal antibody strongly recognized the rhu IL-2 in Western blots, while the chimera was recognized less intensely, likely due to the small IL-2 fragment represented in the recombinant antagonist (see Figure 4B).

### Mass spectrometric analysis

The tryptic peptides ESI-MS spectrum of TH1 antagonist was obtained with a very good agreement between the molecular masses of the predicted proteolytic fragments and the experimental values. A summary of this comparison is shown in Table 2. Three tryptic peptides containing some of the sixty N-terminal amino acids of hu IL-2 (peptides 1–3, Table 2) and the extracellular domain of IFNGR1 were obtained (peptides 5–12, Table 2). The peptide (56A-R66, peptide #4, Table 2) that links both proteins was also detected in the ESI-MS spectrum, confirming that a linker of four amino acids covalently binds these two proteins. The results shown in Table 2, account for 63 % sequence coverage, which is a satisfactory result in the verification of primary structures of proteins extracted from SDS-PAGE. The protein tryptic spectrometric analysis showed the presence of fragments corresponding to the IL-2 N-terminal and IFN-γ extracellular regions confirming the presence of the components of chimeric protein. In order to obtain a further confirmation that this chimeric protein is present in the digested band, the peptides corresponding to the four most intense signals in the ESI-MS spectrum (data not shown) were sequenced. Their ESI-MS/MS spectra were manually interpreted and partial sequences introduced into the PepSea database search program. Peptide 1 (Table 2) is contained in the N-terminal end of hu IL-2 whereas peptides 6, 11 and 12 (Table 2) belong to IFNGR1. These two polypeptides are fused into a single polypeptide chain that migrates as a 33 kDa band as demonstrated by SDS-PAGE (Figure 4A). The aminoacid (in one letter code) sequence of the chimeric TH1 antagonist protein is the following: (bold letters indicated the peptides identified by Mass Spectrometric analysis, underlined is the linking peptide).

MAPTSSSTKK**T_11_QLQLEHLLLDLQMILNGINNYK_33_**NPKLTR**M_40_LTFK_44_F_45_YMPKK_50_**ATE**LKH_56_LQCLAHMMSR_66_A_67_EMGTADLGPSSVPTPTNVTIESYNMNPIVYWEYQ_101_IMPQVPVFTVEVK_114_**NYGVK**N_120_SEWIDACINISHHYCNISDHVGDPSNSWVR_151_**VKARVGQKESAYAK**S_166_EEFAVCR**_173_DGK**I_177_GPPK_181_**LDIRKEEK**Q_190_IMIDIFHPSVFVNGDEQEVDYDPETTCYIR_220_V_221_YNVYVR_227_M_228_NGSEIQYK_236_**ILTQKEDDCDEIQCQLAIPVSSLNSQYCVSAEGVLHVWGVTTEKSKEVCITIFNSSIKG.

The analyzed results are compatible with the recombinant chimeric antagonist protein comprising amino acids 1–60 from human IL-2 N-terminal followed by a peptide followed by a peptide *Ala-Hist-Met-Met *(underline in the above aminoacid sequence) and the 228 aminoacids from the human IFNGR1. This protein contains one cysteine from IL-2 N-terminal and 8 cysteine residues from IFNGR1 extracellular region. For comparison, the chimeric protein sequence with the known IL-2 and IFNGR1 sequences see the following access numbers for IL-2 [NCBI protein: CAA07317] and for IFNGR1 [NCBI protein: CAI21593]. The reported sequences in the PubMed databases include the amino acids sequences corresponding to signal peptide at the beginning of the sequences. It must be taken in account that, the cloned by us sequences correspond to the mature protein without signal peptide.

### TH1 chimeric antagonist biological activities

#### IL-2 antagonist/agonistic action

**Figure 5:**
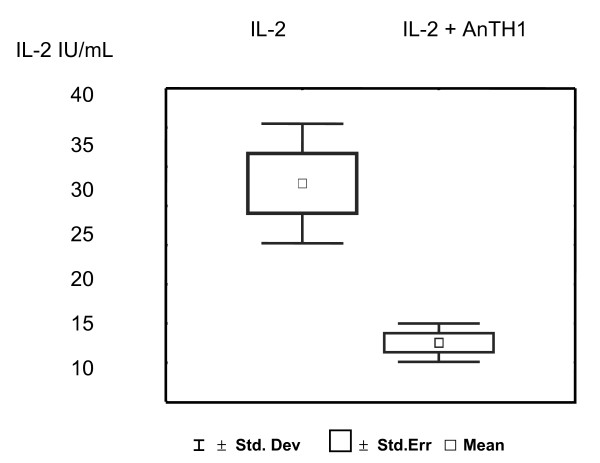
**Inhibition of growth stimulating activity of rhu IL-2 on the mouse cell line CTTL-2 by recombinant chimeric protein**. The data are results from 3 independent experiments. The observed difference proved to be statistically different (p < 0.05) by the Kruskal-Wallis test. IL-2 dilution 1:40 000 correspond to 25 IU/mL (2.8 ng/mL). Group1: IL-2, group 2: IL-2 + TH1 antagonist. Results expressed in international units of IL-2 where 1 IU is defined as the amount of IL-2 that elicits a 50% increase in cell growth in a cell base bioassay.

**Figure 6:**
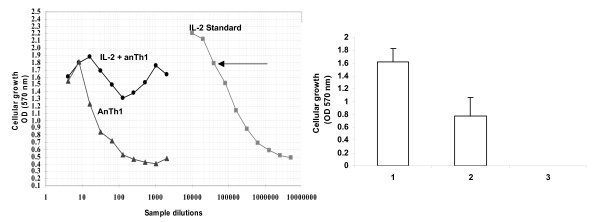
**IL-2 antagonistic/agonistic biological activities of the recombinant chimeric protein**. Panel A: ***Antagonistic/agonistic biological activities***. The figure shows plots of a representative CTLL-2 proliferative assay showing the OD of tested samples in each of the evaluated serial dilutions. The IL-2 used for the experiments is the IL-2 international standard (ST-IL-22010397, 880 000 UI/mL). The arrow shows the OD of IL-2 dilution to test in the presence of serial dilutions of recombinant protein. Panel B: Stimulation of T cells proliferation by TH1 antagonist. *Bar 1*: 2.8 ng/ml of IL-2 (dilution 1:40000), *bar 2*: 1.5 μg/ml of AnTH1, *bar 3*: culture medium without IL-2.

The biological activity of the TH1 antagonist protein was tested in vitro in an assay examining the capacity of the molecule to inhibit the proliferation of T cells in response to rhu IL-2. The descriptive statistic indicates that chimeric protein reduced the activity of IL-2 corresponding to 27.7 IU/mL (median: 28.2, minimum: 20.0, maximum: 34.8 and std. dev 7.6), to 7.5 IU/mL (median 7.9, minimum: 4.2, maximum: 10.0 and std. dev. 2.5), a difference statistically significant with a p = 0.02 (Figure 5 and 6A). The chimera shares also IL-2 agonistic action, as it sustained the growth of murine T cells dependent on exogenous IL-2 (Figure 6A, 6B). In this case, the absorbance measured during the proliferative assay for CTLL-2 cells incubated with 2.8 ng/mL of IL-2 has a mean value of 1.6, while for 1.5 μg/mL of TH1 antagonist molecule the absorbance was approximately the half (Figure 6B).

As mentioned before, the high-affinity receptor for IL-2 is composed of three subunits, IL-2Rα, IL-2Rβ and IL-2Rγ. Moreover, the N-terminal IL-2 region contains all the aminoacids interacting with the IL-2Rα subunit (Lys 35, Arg 38, Phe 42, and Lys 43) and those contacting the IL-2Rβ subunit (Asp 20 and others) [33]. Then the antagonism for the biological activity of IL-2 could be explained by the interaction of IL-2 regions in the fragment of the antagonist described as contacting with the indicated IL-2 receptor subunits. Probably the first sixty aminoacid of N-terminal region of IL-2 can interfere with high-affinity binding of completely mature IL-2 molecule to its membrane receptor. Otherwise, in the absence of IL-2, the 60-aminoacid N-terminal IL-2 fragment from TH1 antagonist chimera might exert the IL-2 agonistic activity. It has been reported that the N-terminal aminoacid stretch (1–30) from IL-2 binds to IL-2Rβ and reproduce some of the IL-2 biological functions, as lymphokine activated killer (LAK) cells activation, induction of IFN-γ production and T cell proliferation stimulation [34,35]. More work is needed to precisely demonstrate its dual effects.

#### TH1 anatagonist neutralization of IFN-γ activities

**Figure 7:**
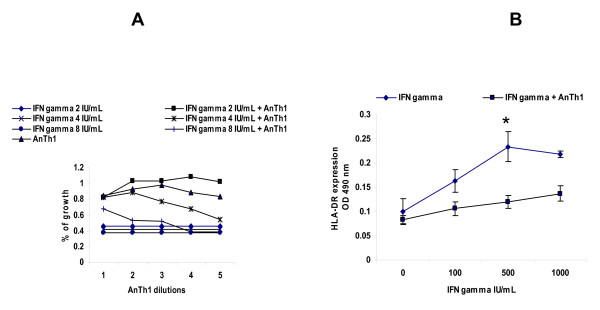
**Neutralization of biological activities of rhu IFN-γ by recombinant chimeric protein**. Panel A: Inhibition of antiproliferative activity of IFN-γ. The inhibition of antiproliferative action of 4 IU/ml of IFN-γ by 50 μg/ml AnTH1 proved to be statistically different (p < 0.05) by the Kruskal-Wallis test. The data are results from 3 independent experiments. The dilutions tested for the antagonist were: 1 (1:4), 2 (1:8), 3 (1:16), 4 (1:32), and 5 (1:64). The OD _580 nm _of cells without IFN-γ at the beginning of the experiment was 0.139 and after 72 hours on culture was 1.068. Panel B: Inhibition of the IFN-γ induced HLA-DR by the AnTh1. The inhibition of IFN-γ (500 IU/mL) HLA-DR stimulation by 1.5 μg/ml AnTh1 proved to be statistically different (p < 0.05) by the Kruskal-Wallis test. The data are the results from 3 independent experiments. The amount of IFN-γ was denoted as IU of antiviral activity, where 1 IU is defined as the inverse of the dilution that conferred 50% protection to the monolayer of cells infected with a virus.

In order to test for inhibition of antiproliferative action of rhu IFN-γ we used HEp-2 cells, known to be highly susceptible to growth inhibition by IFN-γ. During antiproliferative activity assays 2, 4 and 8 ng/ml of IFN-γ inhibited the growth of HEp-2 cells by approximately 60%. Addition of 50 μg/mL of TH1 antagonist to 2 or 4 IU/mL of IFN-γ fully restored proliferation of cells. Neutralization of 8 IU/mL (Figure 7A) was incomplete. The data demonstrated that the antagonist has the capacity to inhibit the antiproliferative action of IFN-γ. However, this effect is obtained at a low molar ratio. This may be due to the high sensitivity of the HEp-2 cell line to growth inhibition of IFN-γ, as evidenced by the data of figure 7, showing that 2 IU/mL of IFN-γ alone had almost a saturating antiproliferative effect on HEp-2 cells.

Treatment of Colo 205 with IFN-γ resulted in the induction of a HLA-DR, a class II MHC antigen, one of the properties of IFN-γ that account for its immunomodulatory action. The stimulation of HLA-DR expression by IFN-γ (0.5 μg/mL) was significantly regulated down (*p = 0.03) in the presence of the TH1 antagonist (1.5 μg/mL) (Figure 7B). Thus, in the experiments designed to test the in vitro biological activities of recombinant TH1 antagonist, the molecule inhibited the antiproliferative and immunomodulatory activities of IFN-γ and the proliferate activity of IL-2. Based on these in vitro biological activities, the antagonist demonstrated a high potential to interfere with the in vivo IL-2 and IFN-γ functions during the activation of the immune system, a characteristic of the inflammatory and autoimmune conditions. Nonetheless, in vivo immune response is essentially normal in mice lacking IL-2 signaling [22]; therefore, the antagonist may not strongly impact TH1 response in regard to IL-2 during early in immune response. However, the antagonist may influence later in the immune response because; IL-2 contributes to T-cell immunity in vivo and seems to be more important during the later stages of immune responses. It has been shown to contribute to the type or magnitude of effectors cells that are produced and to be involved in its migration or proliferation in non-lymphoid tissue.

The TH1 antagonist has other potentially useful characteristics. Beside its capacity of interferes with IL-2, it shows IL-2 agonistic activity, a property that during clinical application might allow to avoid deactivation of the immune system, an adverse effect that is characteristic of current anti-cytokine antagonists therapies, and favors the occurrence of opportunistic infections, demyelization and congestive heart failure [36,37]. Furthermore, because IL-2αR subunit is expressed only in activated T cells, the antagonist might be targeted mainly to this T cell subset or to cells expressing the IL-2βR subunit. Thereby, the agent might be concentrated in the extracellular milieu where activated T cells are abundant.

The agonistic function of the chimera may also offer the opportunity to promote the IL-2 dependent regulatory function of CD4 (+) CD25 (+) T cells, which has been described as critical in the control of autoimmune diseases[22], in the absence of endogenous IL-2 and likely interfere with it in the presence of high endogenous IL-2 levels. Nonetheless, the suppressive efficacy of Tr cells isolated from mice subjected to various treatments correlated closely with suppression of IFN-γ and IL-2 production by the CD25-effector T cells [38]. In vivo confirmation of these potential properties of TH1 antagonist will offer new opportunities to treat several disease conditions accessible with this therapeutic approach.

## Conclusion

We were able to construct and purified a recombinant chimeric antagonist composed by a 60 amino acid fragment of the N-terminal region of human IL-2 fused to the N-terminal of the extracellular region of the alpha subunit of the gamma IFN-γ receptor was obtained. The chimeric protein conserves the physicochemical abilities of their components. This means, the IFN-γ receptor tail is able to recognize the IFN-γ and in correspondence neutralizes two biological activities of IFN-γ, its antiproliferative action on HEp-2 cell line, and the stimulation of HLA-DR in Colo 205 cells. These data evidenced that the construction is compatible with the active conformation of the IFNGR1 extracelluar region. Others experiments confirmed that the IL-2 fragment of 60 aminoacids from the IL-2 N-terminal is in a correct conformation because elicit a classical well recognized property of IL-2, the stimulation of growth of CTTL-2 cell line dependent on IL-2 for growth and survival. The data evidenced and demonstrated that the components are functional in term of their structure as evidenced from the biological activities they have.

## Methods

### Cells

Raji (human Burkitt lymphoma, ATCC: CCL-86) and Jurkat (human acute T-cell leukemia, ATCC: TIB-152) cells were grown in RPMI 1640 supplemented with 10% fetal calf serum (FCS, Gibco BRL, LIFE TECNOLOGIES™) in plastic flasks with gentle agitation at 37°C and 5% CO_2_, as source of poly-A mRNA. Murine T-cells CTLL-2 (cytotoxic T cell, ATCC: TIB-214), human HEp-2 (laryngeal carcinoma, ATCC: CCL23) and Colo 205 (human colorectal adenocarcinoma, ATCC: CLL-222) cell lines were used to evaluate the biological activity of the antagonist. Cells were cultured in RPMI 1640 (CTLL-2, Colo 205) and MEMCANE (HEp-2) containing gentamycin (50 μg/mL) and 10% FCS at 37°C in a humidified 5% CO_2 _environment. *E. coli *cells W3110 P3 were used for pHu (AnTH1) plasmid expression.

### Isolation of cDNA coding for IL-2 N-terminal and IFNGR1 extracellular region

Poly-A mRNA purified from 2 × 10^8 ^Jurkat and Raji cells, respectively, using the MessageMaker^® ^mRNA Isolation System (Invitrogen Life Technologies) was employed for cDNA RT-PCR amplification. 1–2 μg poly-A mRNAs were reverse transcribed with GeneAmp RNA PCR kit (Perkin Elmer Cetus, Norwalk, Conn.) using random hexamers. The specific primer pairs were the following (overlapping nucleotides are underlined): 5'CCATGACCTACTTTCAAGTTCTACAAAG3' and 5'CATCATATGGGTCTAGACACTGAAGATGTTTC3' for the amplification of IL-2 N-terminal, and 5'CCCATATGATGAGCAGGGCTGAGATGGGC3' and 5'GATCCTTATTTTATACTGCTATTGAAAATG3' for the IFNGR1 extracellular region. Primers for the second round PCR were: 5'CCATGGCACC TACTTTCAAGTTCTACAAAG3' and 5'GGATCCTTATTTTATACTGC TATTGAAAATG 3'.

In brief, first-strand DNA was synthesized in a final volume of 20 μL from 1 μg poly-A mRNA in DEPC-H_2_O using 5 mM MgCL_2_, 1 mM dNTP, 1 U/μL Rnase inhibitor, 2.5 U/μL *MuLV *reverse transcriptase, 2.5 μM random hexamers, 0.75 μM primers, and 1X PCR buffer II. The mixture was incubated in a DNA Thermal Cycler (MJ Research, Inn.) at 25°C for 10 minutes, then 1 hour at 42°C followed by 5 minutes at 99°C and then frozen until use. Complementary DNA (10 μl) were mixed as recommended by providers with the appropriately specific primers and reagents (2 mM MgCl_2_, 2.5 U/100 μL *AmpliTaq *DNA polymerase, 0.15 mM specific primers and 1X PCR Buffer II). Amplification started with 5 minutes denaturation at 94°C, followed by 30 PCR cycles. Each cycle consisted of 60 seconds at 94°C for denaturation, 40 seconds for annealing (temperature depended on the primers were used), and 40 seconds at 72°C for extension. Final extension lasted 5 minutes at 72°C. Twenty five percent of the RT-PCR reaction (5 μl) was transferred to a second round reaction mix with the second round primers and reagents in the same concentrations as described for PCR. The second round PCR (25 cycles) was done using the same cycle schedule.

### Construction of the TH1 antagonist expression vector

The pTPV-1 plasmid, supplied by the Biomedical Research Division at CIGB, was employed as expression vector. It carries the *E. coli *tryptophan promoter, the bacteriophage T4 terminator and the ampicillin resistance gene. PTPV-1 vector was digested stepwise with the *Nco*I and *BamH*I enzymes. Second PCR amplified cDNA was digested with the *BamH*I enzyme and processed to eliminate the enzyme and the buffer. Then PCR product was digested with *Nco*I, and processed as described before. Finally, the vector and the amplified gene fragment were ligated using the *T4 ligase *enzyme.

### Sequencing of the pHu (AnTH1) plasmid

The sequencing of pHu (AnTH1) plasmid was done using the *Taq *Dye Deoxy terminator cycle sequencing kit (Applied Biosystems). One μg of pHu (AnTH1) plasmid was used as template and annealed with 10 ng forward or reverse primers, which hybridize with a promoter region of expression vector. After the separation of unincorporated dye terminators and primers, the products were dried in SpeedVac centrifuge, resuspended in loading buffer, heat denatured and immediately loaded on acrylamide gel in an automated DNA sequencer.

### Expression of the recombinant protein

*E. coli *cells, strain W3110P3, containing pHu (AnTH1) plasmid were grown at 37°C in LB medium with 50 μg/mL tryptophan and 100 μg/mL ampicillin until the cells reached an optical density (OD) of 2.0 at 620 nm. Then cells were inoculated in M9 medium with 4% glucose without tryptophan and 50 μg/mL ampicillin to a final OD of 0.25 and grown during 8 hours at 37°C with agitation. Cells were harvested and kept frozen (-20°C) for future processing.

### Extraction of E. coli cells and refolding

To suspend the cells, 10 mL of 10 mM Tris buffer pH 7.2, 1 mM EDTA was added per g of *E. coli *cells. The cells were digested with lysozyme and the pellet from the digestion was washed in a Polytron homogenizer (IKA, Germany) in 50 mM Tris pH 7.2, 1 mM EDTA containing increasing urea concentrations (from 1 to 8 mol/L). Cell suspensions were centrifuged at 12000 rpm at 4°C, 5 min. and supernatants discarded. The pellet from 4 M urea wash was finally extracted in a Polytron homogenizer with 10 mM Tris buffer pH 7.2, 1 mM EDTA containing 8 mol/L urea (extraction buffer). Cell homogenate was centrifuged at 12000 rpm 5 min. 4°C and supernatant decanted and stored at -20°C until refolding. Supernatant from the 8 M urea extraction (150 mL) was loaded on a Sephadex G-100 column (K9/60 (Pharmacia, Uppsala), equilibrated with 50 mM Tris HCL pH 7.4 containing 4 M urea. The elution was performed in the same buffer at 3 mL/minute. Eluted fractions containing the recombinant protein were pooled and dialyzed against a buffer containing 0.1 Tris-HCl, pH 9.0. The dialysis was then continued against phosphate buffered saline (PBS), pH 7.4.

### Ligand-bloting, SDS-PAGE, immuno-blot

For ligand blot, 5-μL fractions from gel filtration chromatography containing folded AnTH1 recombinant protein, were directly applied to nitrocellulose strips and incubated with 10% defatted milk during 2 h at room temperature. After washing with PBS containing 0.05% Tween 20 (PBS-T), the strips were incubated with ^125^-I labed recombinant IFN-γ, without or in the presence unlabed recombinant IFN-γ. Finally, the strips were thrice washed with PBS-T and exposed for autoradiography. For SDS-PAGE the samples were loaded in sample buffer with or without reducing agents. Bands were visualized by Coomassie Blue R-250 (Sigma) staining.

For immunoblotting samples were loaded as for SDS-PAGE. After blotting, nitrocellulose strips were incubated with 10% defatted milk during 2 h at room temperature, washed (as for ligand blot) and incubated with mouse anti-IFNGR1 monoclonal antibody R99 (9 μg/mL) or rabbit polyclonal antibody against human IL-2 protein, washed, incubated with anti-mouse or anti-rabbit (IgG)-peroxidase conjugate in 1% defatted milk and finally washed. Then, the strips were incubated with developing solution (H_2_O_2_, 5 mg/mL, o-phenylendiamine, and 15%).

### Aminoacid sequence. In-gel digestion

An aliquot (0.5 μg) of purified protein was analyzed by SDS-PAGE and reversed-stained with Zn-imidazol [39]. The band was excised and incubated with a 1% citric acid solution during 5 minutes until complete destaining and incubated another 10 minutes in water to remove the excess of chelating agent. The transparent band was additionally cut in approximately one mm^3 ^cubes dehydrated in a 90 % acetonitrile aqueous solution without TFA, and completely dried in a SpeedVac centrifuge. The gels pieces were rehydrated in 20 μL of 50 mM NH_4_HCO_3 _solution containing 12.5 ng of trypsin, sequencing grade (Promega, USA). The in-gel digestion was incubated overnight at 37°C in a thermomixer (Eppendorf, USA). Additionally, 20 μL of 50 mM NH_4_HCO_3 _solution were added and incubated further for 45 min. Tryptic peptides were extracted using ZipTips C18 (Millipore, USA) previously activated and equilibrated as recommended by the manufacturer. Twenty loading cycles were carried out for extracting tryptic peptides. The digest was acidified with formic acid, incubated 45 minutes at room temperature and another twenty loading cycles were achieved. The Ziptips were washed extensively using a 5 % formic acid solution and proteolytic peptides eluted in 2 μL of 60 % acetonitrile containing 1 % formic acid.

### Mass spectrometry

The Electrospray ionization (ESI-MS) mass spectra were acquired using a hybrid quadrupole orthogonal acceleration tandem mass spectrometer QTOF from Micromass (Manchester, UK) fitted with a Z-spray nanoflow electrospray ion source. The mass spectrometer was operated with a source at 80°C and a drying gas flow of 50 L/h. Two μL of the tryptic peptides were loaded onto the borosilicate nanoflow tip and 900 V and 35 V potentials were applied to nanoflow tip and entrance cone, respectively. To obtain information on peptide sequence, the ESI-MS/MS spectra were acquired as described previously [40]. Data acquisition and processing were performed using the MassLynx system (v 3.5) from Micromass. The most intense signals observed in the ESI-MS spectra were sequenced by MS/MS, sequence tags were manually extracted and used to identify the proteins by Peptide Search program [41]. Peptide mass tolerance was 2 Da in order to identify peptides containing deamidated asparagines residues.

### Inhibition of recombinant rhu IL-2 biological activity

The biological activity of rhu IL-2 was assessed as described [42] using IL-2-dependent murine T lymphocyte cell line CTLL-2 [43]. Cells were grown in RPMI-1640 medium containing 1 mM pyruvate, 2 mM L-glutamine, 40 mM HEPES, 100 U/mL penicillin, 50 μg/mL streptomycin, 50 μM 2- mercaptoethanol and 10% FCS supplemented with 8 U/mL rhu IL-2 (Heber Biotec, Havana; 1.2 × 10^7 ^IU/mg). Before use, cells were washed thrice, resuspended in complete culture medium without IL-2 and incubated during 1 h at 37°C in a humidified 5% CO_2 _atmosphere. Cells were suspended at a density of 4 × 10^5 ^cells/mL, and distributed into 96-well microtiter plates (100 μL per well) already containing 100 μL of two-fold serial dilutions of rhu IL-2 or samples, in complete medium. Samples consisted of a constant amount of rhu IL-2 containing serial dilutions of the antagonist. Following 36 h of incubation at 37°C, 20 μL of 5 mg/mL of MTT were added and plates incubated for 4 h in the same environment. Finally, 50 μL of 10% SDS/0.1 N HCl/50% isopropylalcohol solution were added per well, the plates agitated for 1 h at 37°C, and the absorbance readed at 570 nm using a microplate reader (Tecnosuma, Havana). Results were expressed as hu IL-2 units.

### Inhibition of rhu IFN-γ antiproliferative action

HEp-2 at 2.5 × 10^3 ^cells/mL were seeded in 96-well microtiter plates (COSTAR, Cambridge), cultured in MEMCANE containing 50 μg/ml gentamycin and 10% FCS, at 37°C in a humidified 5% CO_2 _environment. Serial dilutions in MEM-CANE medium with 10 % FCS of chimeric recombinant protein samples were tested. Samples were mixed with an equal volume of medium containing appropriated amounts (see figures notes) of rhu IFN-γ (Heber Biotec, Havana; 1.0 × 10^7 ^IU/mg). After adding the samples, monolayers were incubated during 72 h at 37°C, 5 % CO_2_. The amount of growing cells in triplicate cultures at each point was determined by Crystal Violet staining, and absorbance measured at 490 nm using the microplate reader. The result is defined as % of growth as follows:

% of growth = (AT72h-AC0h/AC72h-AC0h) × 100.

AT72h = Absorbance from treated culture at 72 hr.

AC72h = Absorbance from control culture at 72 hr.

AC0h = Absorbance from culture just prior to the addition of IFN.

### Inhibition of HLA expression

The biological activity of TH1 antagonist was assessed further by testing its ability to prevent IFN-γ from inducing expression of HLA-DR. A bio-ELISA assay according to the method of Gibson and colleagues [44] was carried out. Colo 205 cells were grown to confluence in RPMI 1640 containing 10% FCS, trypsinized and seeded in 96-well tissue culture plates at a density of 2.5 × 10^5 ^cells/well in 0.1 mL of RPMI 1640 containing 10% FCS and finally incubated for 12 h at 37°C in 5% CO_2_. Culture media containing the rhu IFN-γ samples and mixtures of rhu IFN-γ and antagonist protein were added in a 0.1 mL volume to the wells containing Colo 205 cells, and then incubated for 1 h at 37°C. Following incubation, the media was removed and wells washed three times with culture media. Fresh culture media (0.2 ml/well) was added and plates incubated for 48 h at 37°C to allow for induction of HLA/DR antigen. Then, the wells were washed with PBS and fixed for 2 min with ice-cold anhydrous ethyl alcohol. After the alcohol was removed, the wells were washed with PBS and incubated for 1 h at room temperature with mouse monoclonal anti-HLA/DR (DAKO, California) antibody diluted in PBS containing 0.5% bovine serum albumin. PBS was used to wash the wells, and peroxidase-labeled goat anti-mouse IgG was added to each well for 1 h at room temperature. The wells were washed three times with PBS and then developed as described for the ELISA plates.

## Authors' contributions

IBR has made substantial contributions to conception and design of chimera and participated in the cloning, expression and purification of the recombinant protein and in the design of biological activities tests. YTR carried out the purification process characterization and the assays for inhibition of biological activities of IFN-γ. EBG carried out sequencing and contribute to evaluation of biological activities. GPR contribute to cloning and genetic construction of recombinant protein. OFB carried out purification and refolding of the recombinant protein. LJG contributed to amino acid sequencing. HJP carried out the assays for the antagonistic/agonist IL-2 activities. PLS has been involved in critically revising the manuscript and supervised the work concerning the biological activity evaluation. All authors read and approved the final manuscript.

## Figures and Tables

**Table 1:** Balance of several processes of AnTH1 solubilization

	**Total protein (mg)**			**% of solubilization**		
**Process Step**	Exp #1	Exp #2	Exp #3	Exp #1	Exp #2	Exp #3

Supernatant 4 M urea wash	1015.2	92.3	246	NE	NE	NE
Pellet 4 M urea wash	1045.2	378	740	NE	NE	NE
Supernatant 8 M urea solubilization	905.8	264	589	86	69.8	79.5
Pellet 8 M urea solubilization	24	16.5	23	NE	NE	NE

% of solubilization: The amount of protien in inclusion bodies in 4 M urea pellet wash solubilized with 8 M urea and present in the supernatant of 8 M urea.

(total protien (mg) supernatant 8 M urea/Total protein (mg) pellet 4 M urea) × 100.

NE: non evaluated.

**Table 2:** ESI-MS analysis of the tryptic peptides derived from the fused-protein

#	Peptide sequencea)	m/z exp.	m/z theor.	Zb)	Abs. Errorc)
1	_11_T-K_33_^d)^	908.81	908.83	3	0.02
2	_40_M-K_44_	639.34	639.35	1	0.01
3	_45_F-K_50_	685.33	685.33	1	0.00
4	_56_A-R_66_^f)^	442.87	442.88	3	0.01
5	_67_A-K_114_	1781.52	1781.54	3	0.02
6	_101_Q-K_114_^d), e)^	807.93	807.95	2	0.02
7	_120_N-R_151_^f)^	921.14	921.17	4	0.03
8	_166_S-R_173_	506.23	506.23	2	0.00
9	_177_I-K_181_	511.31	511.28	1	0.03
10	_190_Q-R_220_^f)^	1225.20	1225.23	3	0.03
11	_221_V-R_227_^d)^	456.74	456.75	2	0.01
12	_228_M-K_236_^d)^	535.24	535.25	2	0.01

a) The numbering in the sequences of peptides is according to the fused-protein sequence shown in the section Results and discussion.

b) Charge state of individual peptides.

c) Indicate the absolute mass difference between the theoretical and experimental molecular masses of the detected peptides.

d) Peptides sequenced by ESI-MS/MS.

e) Peptide originated by the non-specific cleavage of trypsin

f) Peptide containing free cysteine.
